# *fosA3* overexpression with transporter mutations mediates high-level of fosfomycin resistance and silence of *fosA3* in fosfomycin-susceptible *Klebsiella pneumoniae* producing carbapenemase clinical isolates

**DOI:** 10.1371/journal.pone.0237474

**Published:** 2020-08-28

**Authors:** Uthaibhorn Singkham-in, Netchanok Muhummudaree, Tanittha Chatsuwan

**Affiliations:** 1 Department of Microbiology, Faculty of Medicine, Chulalongkorn University, Bangkok, Thailand; 2 Interdisciplinary Program of Medical Microbiology, Graduate School, Chulalongkorn University, Bangkok, Thailand; 3 Antimicrobial Resistance and Stewardship Research Unit, Faculty of Medicine, Chulalongkorn University, Bangkok, Thailand; Panstwowy Instytut Weterynaryjny - Panstwowy Instytut Badawczy w Pulawach, POLAND

## Abstract

The effective treatment of carbapenemase-producing *Klebsiella pneumoniae* infection has been limited and required novel potential agents. Due to the novel drug development crisis, using old antimicrobial agents and combination therapy have been highlighted. This study focused on fosfomycin which inhibits cell wall synthesis and has potential activity on Enterobacteriaceae. We evaluated fosfomycin activity against carbapenemase-producing *K*. *pneumoniae* and characterized fosfomycin resistance mechanisms. Fosfomycin revealed effective activity against only 31.8% of carbapenemase-producing *K*. *pneumoniae* isolates. The major resistance mechanism was FosA3 production. The co-occurrence of FosA3 overexpression with the mutation of *glpT* (or loss of *glpT*) and/or *uhpT* was mediated high-level resistance (MIC>256 mg/L) to fosfomycin. Moreover, *fosA3* silenced in sixteen fosfomycin-susceptible isolates and the plasmid carrying *fosA3* of these isolates increased 32- to 64-fold of fosfomycin MICs in *Escherichia coli* DH5α transformants. The *in vitro* activity of fosfomycin combination with amikacin by checkerboard assay showed synergism and no interaction in six (16.2%) and sixteen isolates (43.3%), respectively. No antagonism of fosfomycin and amikacin was observed. Notably, the silence of *aac (6)’-Ib* and *aphA6* was observed in amikacin-susceptible isolates. Our study suggests that the combination of fosfomycin and amikacin may be insufficient for the treatment of carbapenemase-producing *K*. *pneumoniae* isolates.

## Introduction

*Klebsiella pneumoniae*, a Gram-negative bacilli pathogen causes hospital-acquired infections including lower respiratory tract, urinary tract, and bloodstream infections. Since the emergence of carbapenem-resistant *K*. *pneumoniae* in clinical settings globally, effective treatment options have been limited [1, 2]. Colistin (polymyxin E) is a last potential agent against carbapenem-resistant *K*. *pneumoniae*, but it should be well-inform due to the rise of resistance rate and toxicity [3]. Therefore, infections caused by carbapenem-resistant *K*. *pneumoniae* urgently require novel potential agents for treatment. Nevertheless, the number of novel antibiotics introduced for treatment strongly decline when compared to the emergence of antibiotic resistance. During this crisis, various old antibiotics including fosfomycin which were previously effective, have been reassessed and used [4].

Fosfomycin inhibits the initial step of bacterial cell wall synthesis by covalent binding to UDP-*N*-acetylglucosamine-3-enolpyruvyltransferase (MurA). Fosfomycin is considerably effective against not only *Escherichia coli*, but also other *Enterobacteriaceae* including *K*. *pneumoniae* [4]. However, the range of fosfomycin susceptibility against carbapenem-resistant *K*. *pneumoniae* (39.2% to 66.2%) has been broader than that against carbapenem-resistant *E*. *coli* (83.3% to 100%) [4, 5]. *K*. *pneumoniae* is resistant to fosfomycin by the production of various fosfomycin-modifying enzymes (such as FosA, FosA3, and FosA5) to catalyze fosfomycin. Several *fosA* genes co-carry with carbapenemase genes (*bla*_NDM_, *bla*_OXA-48_, *bla*_IMP_, and *bla*_KPC_) in plasmids resulting in resistance to both fosfomycin and carbapenems [4]. The target alteration by the mutation of MurA particularly at the active site (Cys115 to Asp) strongly confers fosfomycin resistance in *E*. *coli* [6]. Moreover, fosfomycin resistance is also mediated by reduced fosfomycin uptake by alteration of glycerol-3-phosphate (G3P) or glucose-6-phosphate (G6P) transporters (GlpT and UhpT transporters, respectively) [4, 7].

Although fosfomycin activity is variable against carbapenem-resistant *K*. *pneumoniae*, synergy has been observed when combined with carbapenems, colistin, or aminoglycosides [4]. However, the synergism of fosfomycin and aminoglycosides is controversial among different carbapenemase-producing *K*. *pneumoniae*. Fosfomycin and amikacin combination exhibits less synergistic activity against KPC producers than OXA-48 and/or NDM producers [8, 9]. These studies demonstrated that the activity of fosfomycin either alone or combination with aminoglycosides is unpredictable against carbapenem-resistant *K*. *pneumoniae*. In this study, we aimed to determine the activity of fosfomycin alone and in combination with amikacin together with characterization of fosfomycin and amikacin resistance mechanisms among carbapenem-resistant *K*. *pneumoniae* clinical isolates.

## Materials and methods

### Bacterial isolates

A total of 66 carbapenemase-producing *K*. *pneumoniae* which isolated from the patients was obtained from the routine laboratory’s stocks at the King Chulalongkorn Memorial Hospital, Bangkok, Thailand during 2017–2018. This study was approved by the Institutional Review Board of the Faculty of Medicine, Chulalongkorn University (IRB 221/62). Neither human nor animal was involved in this study. The need for consent was waived by the ethics committee.

### Antimicrobial susceptibility testing

Susceptibility testing to imipenem (Apollo Scientific, Manchester, UK) meropenem (Sigma-Aldrich, Steinheim, Germany) and amikacin (Sigma-Aldrich, Steinheim, Germany) was determined by agar dilution method. Fosfomycin (Wako Pure Chemical Industries, Osaka, Japan) susceptibility testing was performed on Mueller-Hinton agar (MHA) (Becton Dickinson BBL, MD, USA) supplemented with 25 mg/L glucose-6-phosphate (G6P) (Sigma-Aldrich, Steinheim, Germany). *E*. *coli* ATCC 25922 and *P*. *aeruginosa* ATCC 27853 were used as reference control strains. Susceptibility of imipenem, meropenem, and fosfomycin was interpreted according to the breakpoints and guidance of the European Committee on Antimicrobial Susceptibility Testing (EUCAST 2020).

### Detection of antimicrobial resistance genes

The metallo-carbapenemase genes (*bla*_IMP-like_ and *bla*_VIM-like_) were detected by multiplex PCR as described previously [10]. Other carbapenemase genes including *bla*_NDM-like_, *bla*_OXA-48-like_, and *bla*_KPC-like_ were detected by another multiplex PCR as described previously [11]. The fosfomycin-modifying enzyme genes (*fosA*, *fosA3*, *fosA5*, *fosB*, *fosC2*, and *fosX*) were detected by PCR using primers listed in S1 Table. The 16S rRNA methylase genes (*armA*, *rmtB*, and *rmtC*) and the aminoglycoside-modifying enzyme (AME) genes (*aac (6)’-Ib* and *aphA6*) were screened by PCR using primers listed in S1 Table.

### Expression of fosfomycin-modifying enzyme genes

Total RNA of *K*. *pneumoniae* was extracted by using Monarch total RNA miniprep kit (NEB, USA) and converted to cDNA by using SuperScript^®^ III reverse transcriptase (Thermo Fisher Scientific, USA). The numbers of fosfomycin-modifying enzyme transcripts were determined by qRT-PCR using Luna^®^ Universal qPCR master mix (NEB, USA) and QuantStudio5 (Thermo Fisher Scientific, USA). Relative expression levels of fosfomycin-modifying enzyme genes were calculated and normalized with 16S rRNA. The qRT-PCR experiments were performed in at least three independent experiments.

### Transformation of the *fosA3*-carrying plasmid into *E*. *coli* DH5α

*K*. *pneumoniae* plasmids were extracted by using HiYield Plasmid Mini Kit (RBC, Taipei, Taiwan) and transformed into *E*. *coli* DH5α by using the heat shock method. The *E*. *coli* DH5α transformants were selected on MHA supplemented with fosfomycin and G6P. The presence of *fosA3* in *E*. *coli* transformants was confirmed by PCR. Fosfomycin susceptibility of *fosA3*-carrying *E*. *coli* transformants was determined by agar dilution.

### Sequence analysis of *murA*, *glpT*, and *uhpT* gene

The entire sequences of *murA*, *glpT*, and *uhpT* were amplified by PCR as described previously and sequenced by using the BigDye Terminator V3.1 cycle sequencing kit from the 1^st^ Base DNA sequencing service, Malaysia. The amino acid substitutions of MurA, GlpT, and UhpT in *K*. *pneumoniae* isolates were compared with those of wild type of *K*. *pneumoniae* isolate K68 from a previous study by Lu et al [12] and deposited in GenBank accession number KT334183, KT334186, and KT334184, respectively.

### The ability of *K*. *pneumoniae* to grow on different carbohydrates

To investigate the ability of *K*. *pneumoniae* to grow in the presence of different carbohydrates, G6P, or glycerol-3-phosphate (G3P) (Sigma-Aldrich, Steinheim, Germany). Briefly, M9 minimal medium liquid supplemented with 0.2% (w/v) G6P or G3P was inoculated with *K*. *pneumoniae* suspension and incubated at 35 °C with shaking for 48 hr. The growth of *K*. *pneumoniae* was determined by measurement of the OD_600nm_ of the cell suspension compared with M9 supplemented with G6P or G3P without bacterial inoculation.

### Checkerboard assay

*In vitro* activity of fosfomycin in combination with amikacin against carbapenemase-producing *K*. *pneumoniae* was performed by checkerboard assay. Briefly, each well of the row in 96-well microtiter plates was contained cation-adjust Mueller-Hinton broth (CAMHB) (Becton Dickinson BBL, MD, USA) with the two-fold dilution of fosfomycin (supplemented with 25 mg/L of G6P). Besides, each well of the column in the plates was contained CAMHB with the two-fold dilution of amikacin. The checkerboard plate was inoculated with *K*. *pneumoniae* and incubated at 35 °C for 18–24 hr. The fractional inhibitory concentration index (FICI) was calculated and interpreted as synergism (FICI≤0.5), no interaction (FICI>0.5–4), and antagonism (FICI>4).

## Results

### Fosfomycin susceptibility of carbapenemase-producing *K*. *pneumoniae*

All *K*. *pneumoniae* exhibited resistance to imipenem and meropenem except isolate KP35 carrying *bla*_IMP-like_ (Table 1). The majority of isolates co-harbored *bla*_NDM-like_ and *bla*_OXA-48-like_ (n = 38, 57.5%) followed by carrying *bla*_NDM-like_ (n = 17, 25.8%), *bla*_OXA-48-like_ (n = 9, 13.7%) and *bla*_IMP-like_ (n = 2, 3.0%), respectively. Neither *bla*_KPC-like_ nor *bla*_VIM-like_ was found in this study. Resistance to fosfomycin was 68.2% (n = 45) of isolates. Among these, 27 isolates (60.0%) co-carried *bla*_NDM-like_ and *bla*_OXA-48-like_, whereas, 9 (20.0%), 8 (17.8%), and one isolate (2.2%) carried *bla*_NDM-like_, *bla*_OXA-48-like_ and *bla*_IMP-like_, respectively.

**Table 1:** Antimicrobial susceptibility of 66 carbapenemase-producing *K*. *pneumoniae* isolates.

Strain	MIC (mg/L)				Carbapenemase gene	Fos gene	Strain	MIC (mg/L)				Carbapenemase gene	Fos gene
	IPM	MEM	FOF	AK				IPM	MEM	FOF	AK		
**KP35**	2	2	32	1	*bla*_IMP-like_	*fosA5*	**KP12**	32	128	32	16	*bla*_NDM-like_, *bla*_OXA-48-like_	*fosA5*, *fosA3*
**KP71**	32	16	128	1	*bla*_IMP-like_	*fosA5*	**KP21**	32	64	32	16	*bla*_NDM-like_, *bla*_OXA-48-like_	*fosA5*, *fosA3*
**KP79**	32	32	8	8	*bla*_NDM-like_	*fosA5*	**KP30**	64	128	32	16	*bla*_NDM-like_, *bla*_OXA-48-like_	*fosA5*, *fosA3*
**KP23**	16	128	16	16	*bla*_NDM-like_	*fosA5*, *fosA3*	**KP39**	64	128	32	16	*bla*_NDM-like_, *bla*_OXA-48-like_	*fosA5*, *fosA3*
**KP97**	16	16	16	2	*bla*_NDM-like_	*fosA5*, *fosA3*	**KP81**	64	128	32	1	*bla*_NDM-like_, *bla*_OXA-48-like_	*fosA5*, *fosA3*
**KP53**	16	32	32	8	*bla*_NDM-like_	*fosA5*	**KP90**	32	64	32	2	*bla*_NDM-like_, *bla*_OXA-48-like_	*fosA5*, *fosA3*
**KP72**	32	16	32	16	*bla*_NDM-like_	*fosA5*	**KP3**	32	64	64	16	*bla*_NDM-like_, *bla*_OXA-48-like_	*fosA5*, *fosA3*
**KP78**	128	32	32	16	*bla*_NDM-like_	*fosA5*	**KP32**	32	64	64	8	*bla*_NDM-like_, *bla*_OXA-48-like_	*fosA5*, *fosA3*
**KP14**	8	32	32	16	*bla*_NDM-like_	*fosA5*, *fosA3*	**KP45**	64	128	64	16	*bla*_NDM-like_, *bla*_OXA-48-like_	*fosA5*, *fosA3*
**KP68**	64	128	32	16	*bla*_NDM-like_	*fosA5*, *fosA3*	**KP46**	64	128	64	2	*bla*_NDM-like_, *bla*_OXA-48-like_	*fosA5*, *fosA3*
**KP34**	128	128	64	256	*bla*_NDM-like_	*fosA5*	**KP49**	64	128	64	16	*bla*_NDM-like_, *bla*_OXA-48-like_	*fosA5*, *fosA3*
**KP48**	32	64	64	16	*bla*_NDM-like_	*fosA5*, *fosA3*	**KP52**	64	256	64	16	*bla*_NDM-like_, *bla*_OXA-48-like_	*fosA5*, *fosA3*
**KP54**	32	64	64	1	*bla*_NDM-like_	*fosA5*, *fosA3*	**KP60**	>256	256	64	4	*bla*_NDM-like_, *bla*_OXA-48-like_	*fosA5*, *fosA3*
**KP61**	64	128	64	16	*bla*_NDM-like_	*fosA5*, *fosA3*	**KP73**	32	128	64	16	*bla*_NDM-like_, *bla*_OXA-48-like_	*fosA5*, *fosA3*
**KP64**	64	128	64	16	*bla*_NDM-like_	*fosA5*, *fosA3*	**KP95**	64	128	64	>256	*bla*_NDM-like_, *bla*_OXA-48-like_	*fosA5*, *fosA3*
**KP87**	128	128	64	16	*bla*_NDM-like_	*fosA5*, *fosA3*	**KP36**	128	256	128	128	*bla*_NDM-like_, *bla*_OXA-48-like_	*fosA5*
**KP70**	16	32	128	8	*bla*_NDM-like_	*fosA5*, *fosA3*	**KP94**	>256	256	128	2	*bla*_NDM-like_, *bla*_OXA-48-like_	*fosA5*
**KP82**	16	16	128	16	*bla*_NDM-like_	*fosA5*, *fosA3*	**KP57**	64	128	128	16	*bla*_NDM-like_, *bla*_OXA-48-like_	*fosA5*, *fosA3*
**KP58**	256	256	>256	16	*bla*_NDM-like_	*fosA5*, *fosA3*	**KP84**	64	128	256	16	*bla*_NDM-like_, *bla*_OXA-48-like_	*fosA5*, *fosA3*
**KP44**	4	16	32	8	*bla*_OXA-48-like_	*fosA5*, *fosA3*	**KP4**	32	64	>256	16	*bla*_NDM-like_, *bla*_OXA-48-like_	*fosA5*, *fosA3*
**KP27**	128	128	64	256	*bla*_OXA-48-like_	*fosA5*	**KP6**	32	64	>256	16	*bla*_NDM-like_, *bla*_OXA-48-like_	*fosA5*, *fosA3*
**KP29**	4	4	128	8	*bla*_OXA-48-like_	*fosA5*, *fosA3*	**KP15**	32	64	>256	16	*bla*_NDM-like_, *bla*_OXA-48-like_	*fosA5*, *fosA3*
**KP62**	16	32	>256	>256	*bla*_OXA-48-like_	*fosA5*	**KP18**	256	256	>256	16	*bla*_NDM-like_, *bla*_OXA-48-like_	*fosA5*, *fosA3*
**KP11**	8	32	>256	>256	*bla*_OXA-48-like_	*fosA5*	**KP19**	>256	256	>256	32	*bla*_NDM-like_, *bla*_OXA-48-like_	*fosA5*, *fosA3*
**KP26**	8	32	>256	>256	*bla*_OXA-48-like_	*fosA5*	**KP43**	32	64	>256	8	*bla*_NDM-like_, *bla*_OXA-48-like_	*fosA5*, *fosA3*
**KP63**	8	32	>256	>256	*bla*_OXA-48-like_	*fosA5*, *fosA3*	**KP47**	32	128	>256	16	*bla*_NDM-like_, *bla*_OXA-48-like_	*fosA5*, *fosA3*
**KP88**	64	256	>256	32	*bla*_OXA-48-like_	*fosA5*, *fosA3*	**KP50**	64	128	>256	16	*bla*_NDM-like_, *bla*_OXA-48-like_	*fosA5*, *fosA3*
**KP51**	8	32	>256	>256	*bla*_OXA-48-like_	*fosA5*, *fosA3*	**KP55**	64	128	>256	16	*bla*_NDM-like_, *bla*_OXA-48-like_	*fosA5*, *fosA3*
**KP31**	16	8	16	8	*bla*_NDM-like_, *bla*_OXA-48-like_	*fosA5*, *fosA3*	**KP56**	128	128	>256	1	*bla*_NDM-like_, *bla*_OXA-48-like_	*fosA5*, *fosA3*
**KP1**	32	64	32	16	*bla*_NDM-like_, *bla*_OXA-48-like_	*fosA5*, *fosA3*	**KP59**	128	128	>256	16	*bla*_NDM-like_, *bla*_OXA-48-like_	*fosA5*, *fosA3*
**KP2**	32	64	32	16	*bla*_NDM-like_, *bla*_OXA-48-like_	*fosA5*, *fosA3*	**KP89**	>256	256	>256	32	*bla*_NDM-like_, *bla*_OXA-48-like_	*fosA5*, *fosA3*
**KP5**	32	64	32	16	*bla*_NDM-like_, *bla*_OXA-48-like_	*fosA5*, *fosA3*	**KP92**	32	128	>256	16	*bla*_NDM-like_, *bla*_OXA-48-like_	*fosA5*, *fosA3*
**KP8**	32	64	32	16	*bla*_NDM-like_, *bla*_OXA-48-like_	*fosA5*, *fosA3*	**KP7**	64	128	>256	16	*bla*_NDM-like_, *bla*_OXA-48-like_	*fosA5*, *fosA3*

IPM: Imipenem; MEM: Meropenem; FOF: Fosfomycin; AK: Amikacin

### Prevalence and mRNA expression level of fosfomycin-modifying enzyme genes

Intrinsic fosfomycin-modifying enzyme gene, *fosA5* was found in all *K*. *pneumoniae* isolates (Table 1). Among forty-five fosfomycin-resistant isolates, 37 isolates (82.2%) carried *fosA3* with fosfomycin MIC range of 64–>256 mg/L. Interestingly, the silence of *fosA3* was also present in 16 fosfomycin-susceptible isolates with the MIC range of 16–32 mg/L.

**Fig 1:**
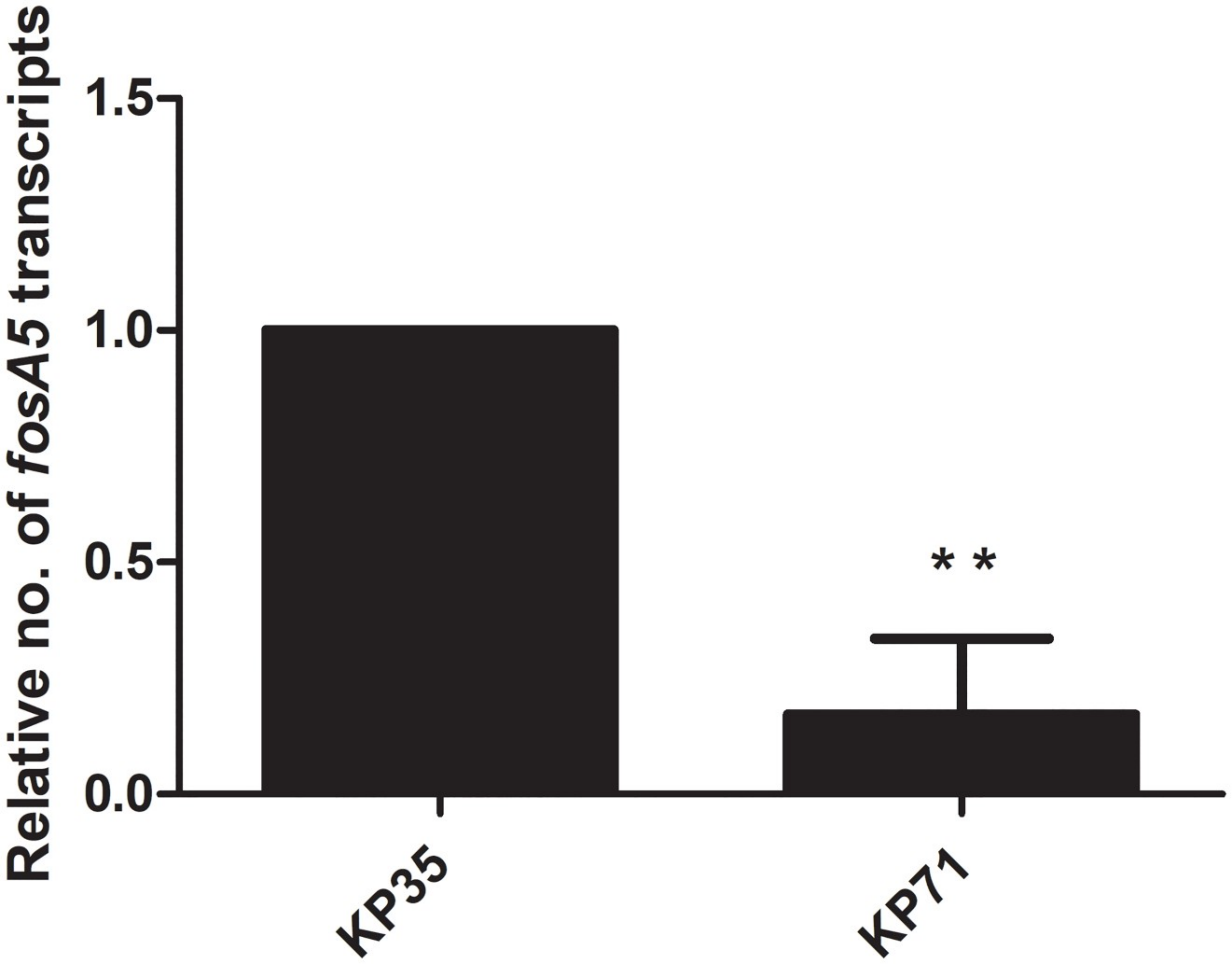
Relative *fosA5* expression levels of IMP-producing *K*. *pneumoniae*. qRT-PCR assay of *fosA5* expression was performed in *K*. *pneumoniae* isolate KP35 and KP71. The relative number of transcripts of *fosA5* were normalized to 16S rRNA expression and calculated using the 2^-ΔΔct^ method compared to the expression of fosfomycin-susceptible *K*. *pneumoniae* isolate KP35. *p*-values were calculated using unpaired t-test (*, *p*-value <0.05; **, *p*-value <0.01; ***, *p*-value <0.001 and ns, non-significant).

**Fig 2:**
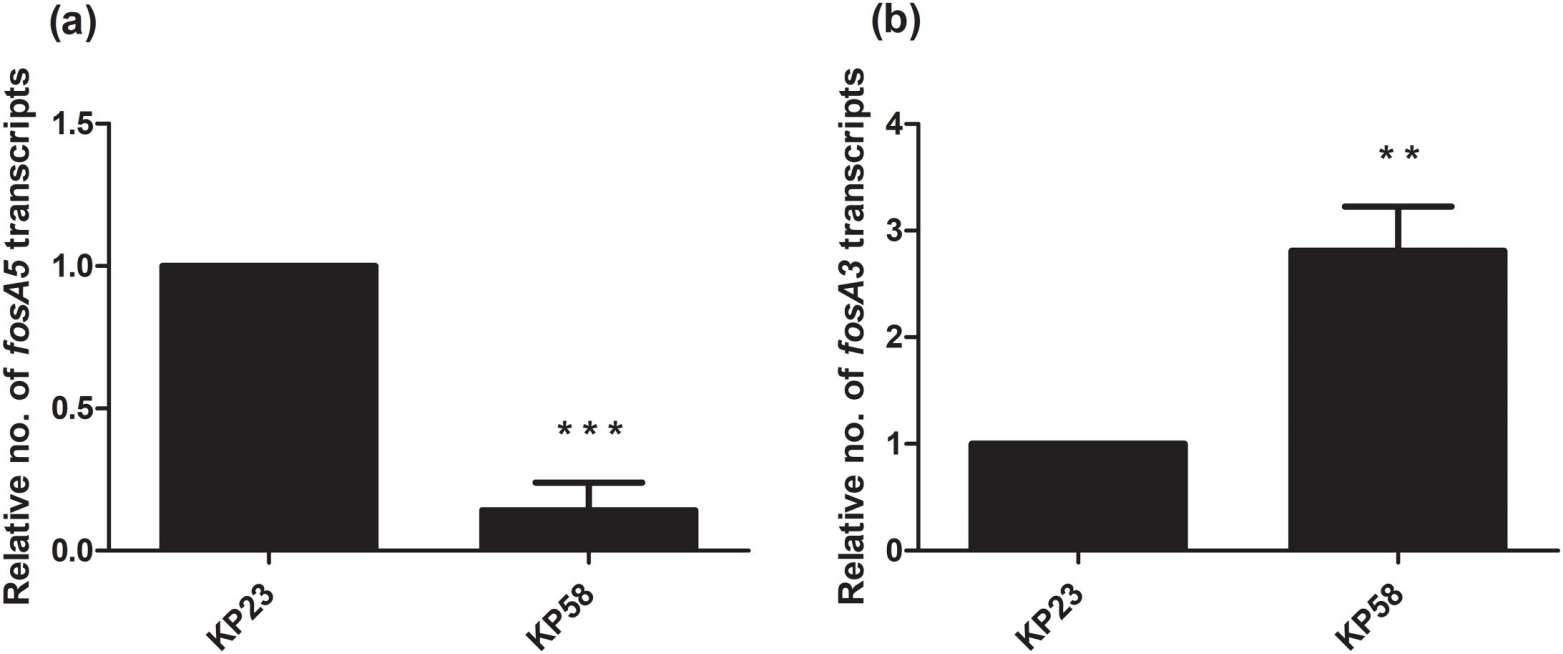
Relative *fosA5* and *fosA3* expression levels of NDM-producing *K*. *pneumoniae*. qRT-PCR assay of *fosA5* (a) and *fosA3* (b) expression was performed in *K*. *pneumoniae* isolate KP23 and KP58. The relative number of transcripts of *fosA5* and *fosA3* were normalized to 16S rRNA expression and calculated using the 2^-ΔΔct^ method compared to the expression of fosfomycin-susceptible *K*. *pneumoniae* isolate KP23. *p*-values were calculated using unpaired t-test (*, *p*-value <0.05; **, *p*-value <0.01; ***, *p*-value <0.001 and ns, non-significant).

**Fig 3:**
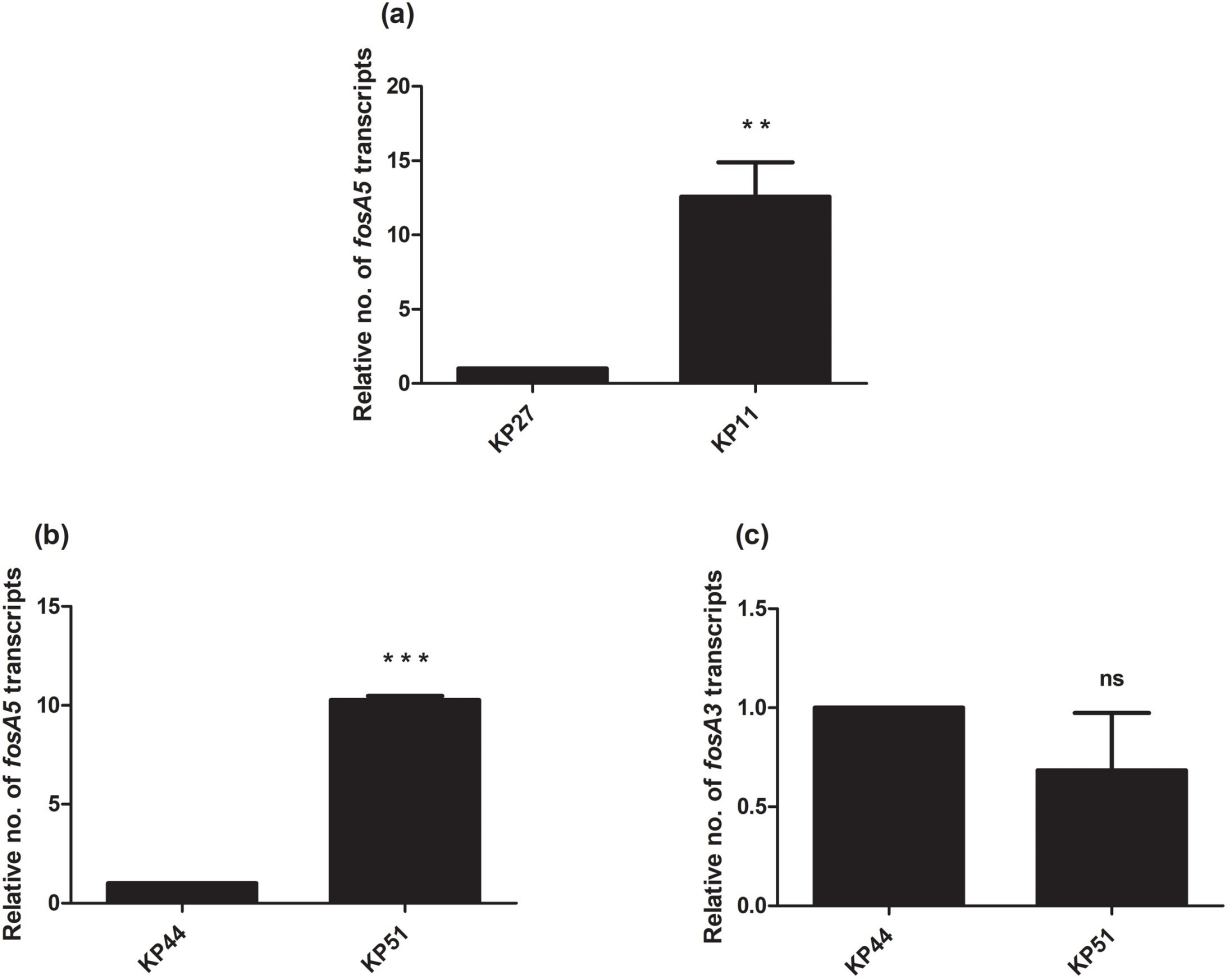
Relative *fosA5* and *fosA3* expression levels of OXA-48-producing *K*. *pneumoniae*. qRT-PCR assay of *fosA5* (a) and *fosA3* (b, c) expression was performed in *K*. *pneumoniae* isolate KP27, KP11, KP44, and KP51. The relative number of transcripts of *fosA5* and *fosA3* were normalized to 16S rRNA expression and calculated using the 2^-ΔΔct^ method compared to the expression of fosfomycin-susceptible *K*. *pneumoniae* isolate KP27 or KP44. *p*-values were calculated using unpaired t-test (*, *p*-value <0.05; **, *p*-value <0.01; ***, *p*-value <0.001 and ns, non-significant).

**Fig 4:**
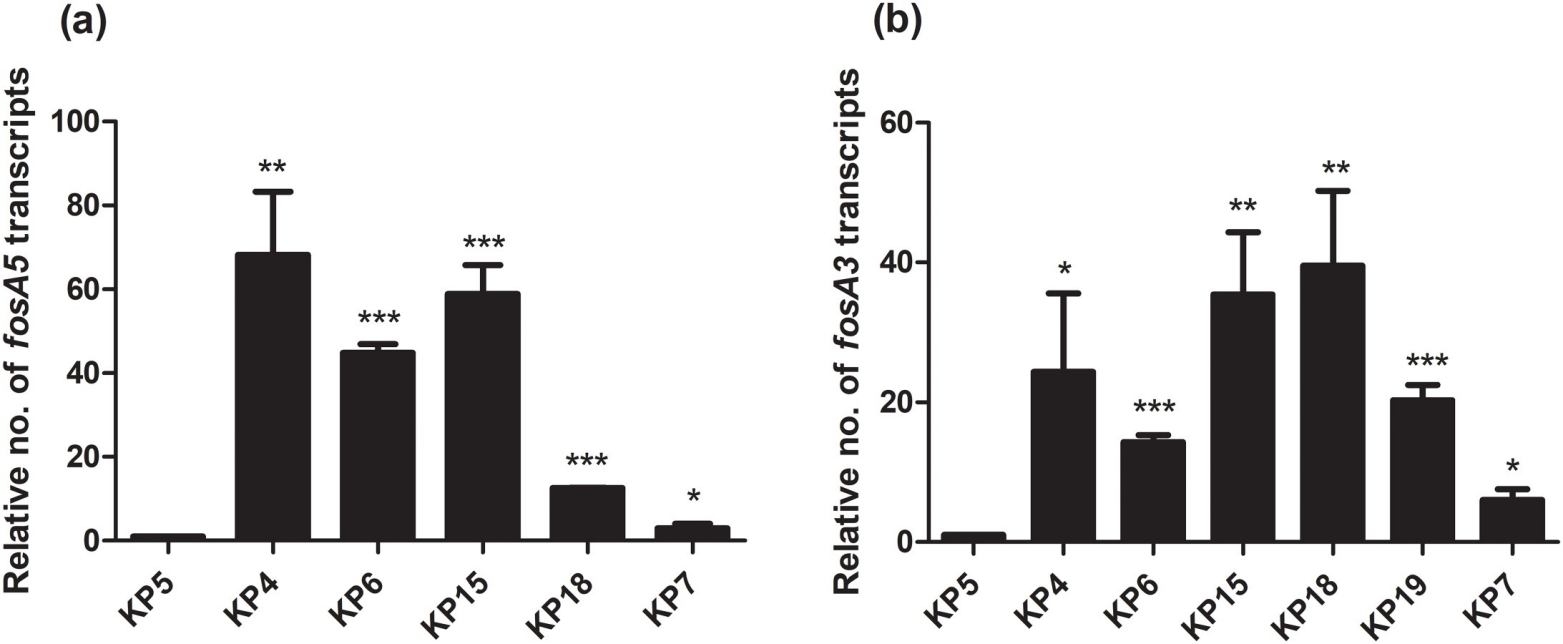
Relative *fosA5* and *fosA3* expression levels of NDM and OXA-48-coproducing *K*. *pneumoniae*. qRT-PCR assay of *fosA5* (a) and *fosA3* (b) expression was performed in *K*. *pneumoniae* isolate KP5, KP4, KP6, KP15, KP18, KP19, and KP7. The relative number of transcripts of *fosA5* and *fosA3* were normalized to 16S rRNA expression and calculated using the 2^-ΔΔct^ method compared to the expression of fosfomycin-susceptible *K*. *pneumoniae* isolate KP5. *p*-values were calculated using unpaired t-test (*, *p*-value <0.05; **, *p*-value <0.01; ***, *p*-value <0.001 and ns, non-significant).

Due to the presence of *fosA5* and *fosA3* in *K*. *pneumoniae* with different fosfomycin MICs, we determined the relationship of expression levels of *fosA5* and *fosA3* and fosfomycin susceptibility among each group of carbapenemase production. In IMP-producing strains, interestingly, *fosA5* expression of fosfomycin-resistant strain (KP71, MIC = 128 mg/L) was lower than that of KP35 which was susceptible to fosfomycin (Fig 1). In NDM-producing isolates, *fosA3* expression of fosfomycin-resistant isolate (KP58, MIC = >256 mg/mL) was significantly higher than that of susceptible isolate (Fig 2). In the case of NDM producers, fosfomycin-resistant isolates (KP11 and KP51) showed higher *fosA3* expression levels but a similar level of *fosA5* expression to fosfomycin-susceptible isolates (Fig 3). In contrast, fosfomycin-resistant strains with co-producing NDM and OXA-48 revealed a strongly higher expression level of *fosA5* as well as *fosA3* (Fig 4).

### Impact of *fosA3* on fosfomycin susceptibility

The fosfomycin MICs of *E*. *coli* DH5α transformants carrying *fosA3* plasmids were evaluated. The transformants carrying *fosA3* plasmid from fosfomycin-susceptible isolates (KP23, KP44, and KP5) revealed 32- to 64-fold increased fosfomycin MICs (Table 2). Moreover, fosfomycin MICs of the transformants receiving *fosA3* plasmid from resistant strains exhibited a 64-fold to >512-fold increase in MICs from the baseline.

**Table 2:** Antimicrobial susceptibility of *K*. *pneumoniae* clinical isolates and *E*. *coli* DH5α carrying *fosA3*.

Strain		MIC (mg/L)	
	Fosfomycin	Imipenem	Meropenem
KP23	16	16	128
KP58	>256	256	256
KP44	32	4	16
KP51	>256	8	32
KP5	32	32	64
KP4	>256	32	64
KP6	>256	32	64
KP15	>256	32	64
KP18	>256	256	256
KP19	>256	>256	256
KP7	>256	64	128
DH5α	0.5	0.25	0.015
DH5α/*fosA3*_KP23	32	0.25	0.015
DH5α/*fosA3*_KP58	128	0.25	0.015
DH5α/*fosA3*_KP44	16	0.25	0.015
DH5α/*fosA3*_KP51	>256	0.5	0.03
DH5α/*fosA3*_KP5	32	0.25	0.015
DH5α/*fosA3*_KP4	32	0.25	0.015
DH5α/*fosA3*_KP6	128	0.5	0.015
DH5α/*fosA3*_KP15	128	0.25	0.03
DH5α/*fosA3*_KP18	64	0.25	0.03
DH5α/*fosA3*_KP19	128	0.25	0.015
DH5α/*fosA3*_KP7	256	0.5	0.03

### Amino acid substitutions in MurA, GlpT, and UhpT

**Fig 5:**
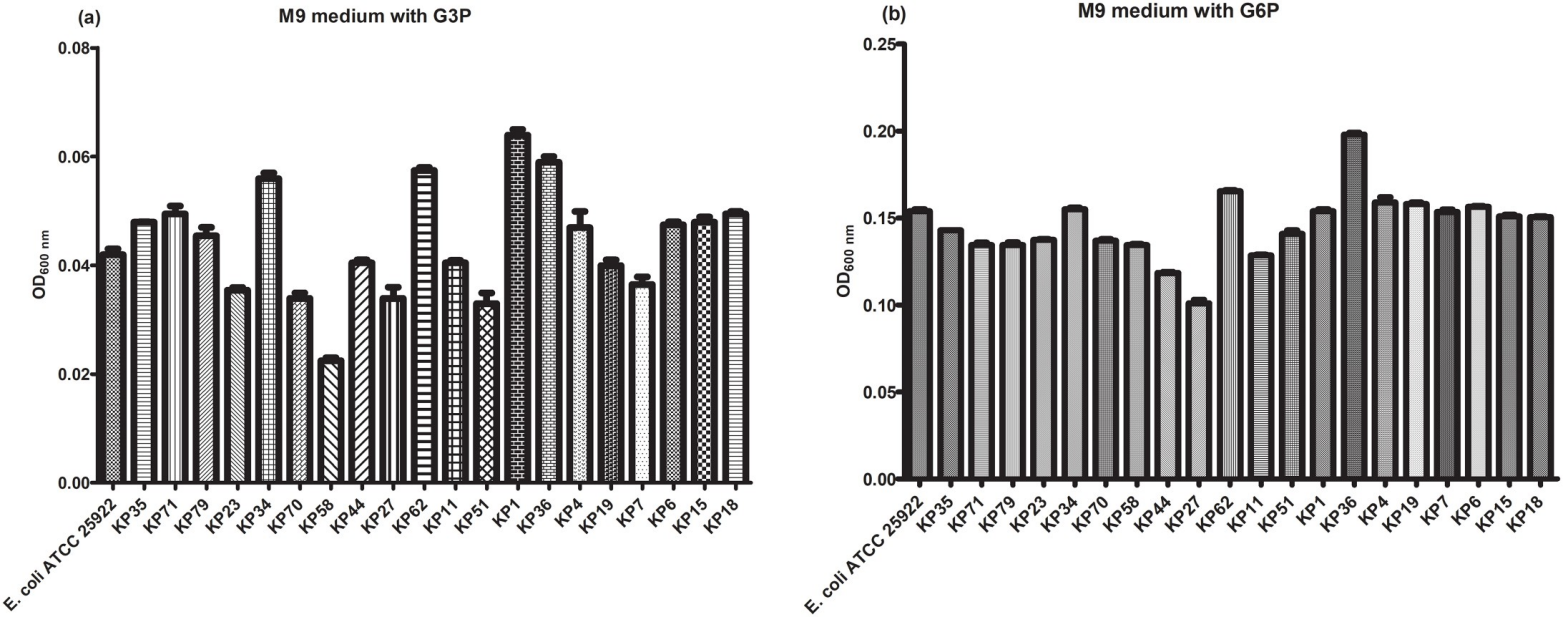
Growth of *K*. *pneumoniae* isolates in M9 minimal medium supplemented with G3P or G6P as a carbon source. The ability of *K*. *pneumoniae* growth was determined by measurement of the OD_600nm_ of the cell suspension, normalized to the OD_600nm_ of M9 supplemented with G6P or G3P without bacterial inoculation and compared to growth ability of *E*. *coli* ATCC 25922.

Although *fosA3* plasmids of *K*. *pneumoniae* had an impact on fosfomycin MIC in *E*. *coli* transformants, the prevalence of *fosA3* among fosfomycin-resistant *K*. *pneumoniae* was only 82.2% indicating that other mechanisms may involve in fosfomycin resistance. Amino acid sequences of MurA, GlpT, and UhpT were characterized in 37 isolates. The Thr287Asn substitution in MurA and Arg171Gly in UhpT were detected in both susceptible and resistant isolates (Table 3). Absence of *glpT* gene was detected in two fosfomycin-resistant isolates (KP58 and KP19) and these isolates showed reduced growth in M9 medium supplemented with G3P (compare to KP70 and KP4, respectively) suggesting an impair function of GlpT transporter (Fig 5). Various amino acid substitutions in GlpT (His147Gln, Pro212Leu, Gly386Ser, Gly386Ile, Phe112Ser, and Pro97Arg) and absence of *glpT* gene were detected in fosfomycin-resistant isolates. However, isolates carrying Gly386Ser, Gly386Ile, Phe112Ser, or Pro97Arg substitutions or absence of GlpT showed decreased growth in the presence of G3P as a carbon source (compare to KP70, KP27, and KP1, respectively). There were other substitutions in UhpT detected in highly resistant isolates including Ala176Pro in KP58 and Leu132Val in KP62 but their growth in the presence of G6P was similar to that of fosfomycin-susceptible isolate KP70 and KP27, respectively. Our results suggest that the overexpression level of *fosA3* with the mutation or lack of *glpT* may be responsible for high-level resistance to fosfomycin.

**Table 3:** Fosfomycin resistance mechanisms of carbapenemase-producing *K*. *pneumoniae*.

Strain	Fosfomycin MIC (mg/L)	Fos gene	Amino acid substitution		
			MurA	GlpT	UhpT
**IMP-producing strain**					
KP35	32	*fosA5*	Thr287Asn	none	Arg171Gly
KP71	128	*fosA5*	Thr287Asn	none	Arg171Gly
**NDM-producing strain**					
KP79	8	*fosA5*	Thr287Asn	none	Arg171Gly
KP23	16	*fosA5*, *fosA3*	Thr287Asn	none	Arg171Gly
KP97	16	*fosA5*, *fosA3*	Thr287Asn	none	Arg171Gly
KP53	32	*fosA5*	Thr287Asn	none	Arg171Gly
KP72	32	*fosA5*	Thr287Asn	none	Arg171Gly
KP78	32	*fosA5*	Thr287Asn	none	Arg171Gly
KP14	32	*fosA5*, *fosA3*	Thr287Asn	none	Arg171Gly
KP68	32	*fosA5*, *fosA3*	Thr287Asn	none	Arg171Gly
KP34	64	*fosA5*	Thr287Asn	none	Arg171Gly
KP70	128	*fosA5*, *fosA3*	Thr287Asn	none	Arg171Gly
KP58	>256	*fosA5*, *fosA3*	Thr287Asn	Not detected	Arg171Gly, Ala176Pro
**OXA-48-producing strain**					
KP44	32	*fosA5*, *fosA3*	Thr287Asn	none	Arg171Gly
KP27	64	*fosA5*	Thr287Asn	none	Arg171Gly
KP62	>256	*fosA5*	Thr287Asn	none	Leu132Val, Arg171Gly
KP11	>256	*fosA5*	Thr287Asn	His147Gln	Arg171Gly
KP26	>256	*fosA5*	Thr287Asn	His147Gln	Arg171Gly
KP63	>256	*fosA5*, *fosA3*	Thr287Asn	Pro212Leu	Arg171Gly
KP51	>256	*fosA5*, *fosA3*	Thr287Asn	Pro212Leu	Arg171Gly
**NDM and OXA-48-coproducing strain**					
KP1	32	*fosA5*, *fosA3*	Thr287Asn	none	Arg171Gly
KP2	32	*fosA5*, *fosA3*	Thr287Asn	none	Arg171Gly
KP5	32	*fosA5*, *fosA3*	Thr287Asn	none	Arg171Gly
KP8	32	*fosA5*, *fosA3*	Thr287Asn	none	Arg171Gly
KP12	32	*fosA5*, *fosA3*	Thr287Asn	none	Arg171Gly
KP21	32	*fosA5*, *fosA3*	Thr287Asn	none	Arg171Gly
KP30	32	*fosA5*, *fosA3*	Thr287Asn	none	Arg171Gly
KP39	32	*fosA5*, *fosA3*	Thr287Asn	none	Arg171Gly
KP81	32	*fosA5*, *fosA3*	Thr287Asn	none	Arg171Gly
KP90	32	*fosA5*, *fosA3*	Thr287Asn	none	Arg171Gly
KP36	128	*fosA5*	Thr287Asn	none	Arg171Gly
KP4	>256	*fosA5*, *fosA3*	Thr287Asn	Gly386Ser	Arg171Gly
KP6	>256	*fosA5*, *fosA3*	Thr287Asn	none	Arg171Gly
KP15	>256	*fosA5*, *fosA3*	Thr287Asn	Gly386Ile	Arg171Gly
KP18	>256	*fosA5*, *fosA3*	Thr287Asn	Phe112Ser	Arg171Gly
KP19	>256	*fosA5*, *fosA3*	Thr287Asn	Not detected	Arg171Gly
KP7	>256	*fosA5*, *fosA3*	Thr287Asn	Pro97Arg	Arg171Gly

### The activity of fosfomycin and amikacin combination

Amikacin alone was less effective against carbapenemase-producing *K*. *pneumoniae* than fosfomycin alone (Table 1). Among 66 isolates, only 18 (27.3%) were susceptible to amikacin. Resistance to amikacin was unlikely related to fosfomycin resistance. High-level resistance to amikacin (MIC>256 mg/L) was observed in OXA-48-producing isolates and NDM with OXA-48-co-producing isolates. However, high-level resistance to both amikacin and fosfomycin was found in OXA-48-producing isolates. The most common aminoglycoside-modifying enzyme gene, *aac (6)’-Ib* was detected in *K*. *pneumoniae* isolates with amikacin MIC range of 2–>256 mg/L (Table 3). Highly amikacin-resistant strains (KP34, KP27, and KP36) also carried *armA*.

**Fig 6:**
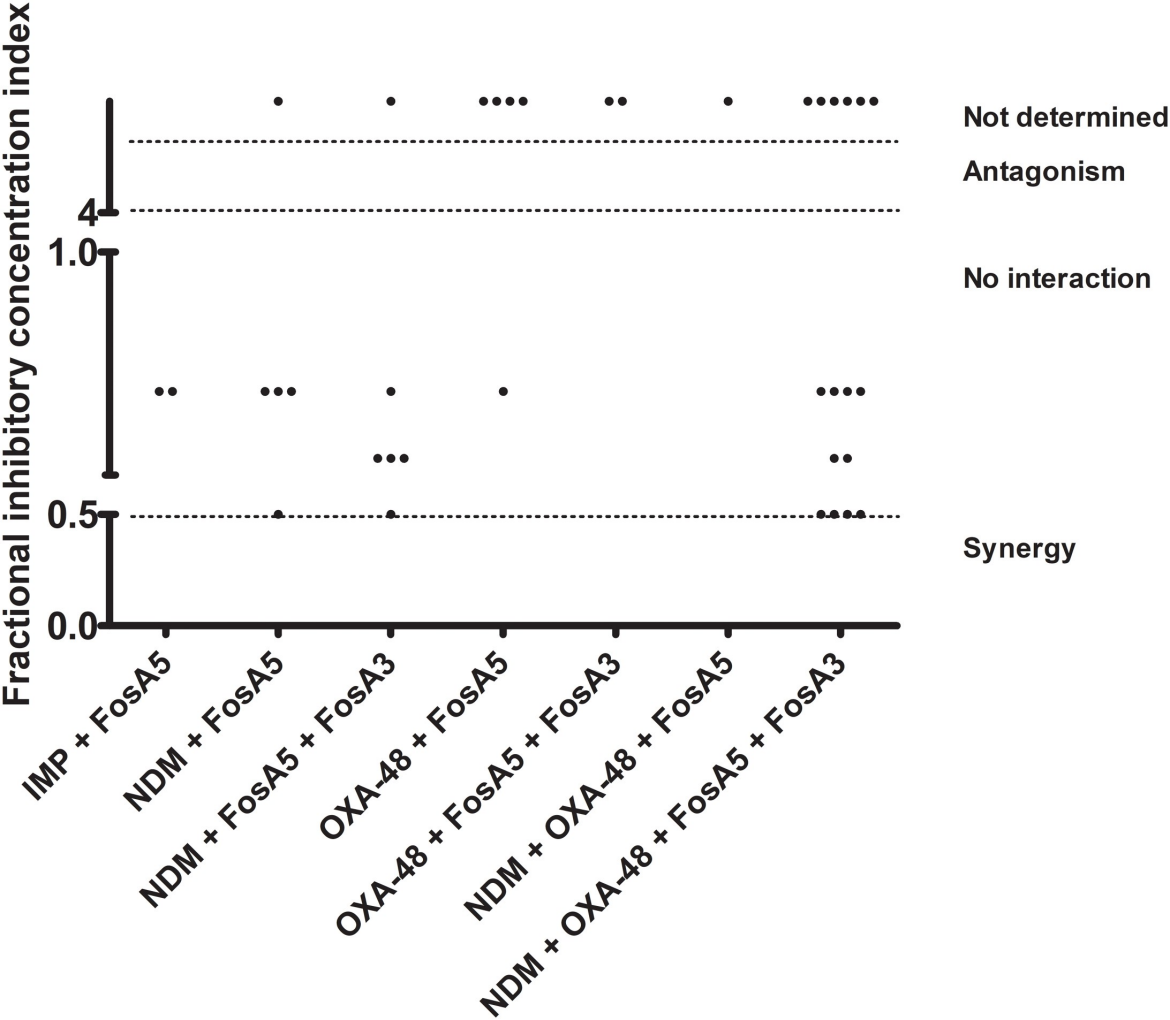
Distribution of Fraction Inhibitory Concentration Index (FICI) of *K*. *pneumoniae* isolates. The FICIs of *K*. *pneumoniae* were plotted with different carbapenemase and *fosA* genes and categorized by synergism (FICI≤0.5), no interaction (0.5<FICI≤4), antagonism (FICI>4) and not determined for isolates with fosfomycin and/or amikacin MIC >256 mg/L.

*In vitro* activity of fosfomycin combination with amikacin was performed in 37 carbapenemase-producing isolates by checkerboard assay. The FICIs of this combination are shown in Table 4 and Fig 6. The synergistic effect was observed in only 6 isolates (16.2%) including two isolates producing NDM and four isolates producing NDM with OXA-48. Fifteen isolates with high-level resistance to either fosfomycin or amikacin (MIC>256 mg/L) were excluded from the synergistic study by checkerboard assay. However, fosfomycin and amikacin MICs of these isolates were only two-fold reduced when compared to those of each antibiotic alone. Moreover, the synergism of fosfomycin and amikacin did not correlate with the presence of aminoglycoside-modifying enzymes. Our results suggest that amikacin unlikely enhances fosfomycin activity against carbapenemase-producing *K*. *pneumoniae*. Fortunately, antagonism was not found in our study.

**Table 4:** The activity of amikacin in combination with fosfomycin against carbapenemase-producing *K*. *pneumoniae*.

Strain	Fosfomycin MIC (mg/L)	Amikacin MIC (mg/L)	Aminoglycoside-modifying enzyme gene	Fosfomycin MIC in combination (mg/L)	Amikacin MIC in combination (mg/L)	FICI
**IMP-producing strain**						
KP35	32	1	NF	16	0.25	0.75
KP71	128	1	NF	64	0.25	0.75
**NDM-producing strain**						
KP79	8	8	*aac (6)’-Ib*	4	2	0.75
KP23	16	16	*aac (6)’-Ib*	8	4	0.75
KP97	16	2	*aac (6)’-Ib*, *aphA6*	2	1	0.63
KP53	32	8	*aac (6)’-Ib*, *aphA6*	8	2	0.75
KP72	32	16	*aac (6)’-Ib*, *aphA6*	8	4	0.5
KP78	32	16	*aac (6)’-Ib*, *aphA6*	8	8	0.75
KP14	32	16	*aac (6)’-Ib*	4	8	0.63
KP68	32	16	*aac (6)’-Ib*	4	8	0.63
KP34	64	>256	*aac (6)’-Ib*, *aphA6*, *armA*	16	256	ND
KP70	128	8	*aac (6)’-Ib*	32	2	0.5
KP58	>256	16	*aac (6)’-Ib*	>256	16	ND
**OXA-48-producing strain**						
KP44	32	8	*aac (6)’-Ib*	8	4	0.75
KP27	64	>256	*aac (6)’-Ib*, *aphA6*, *armA*	16	256	ND
KP62	>256	>256	*aac (6)’-Ib*	>256	>256	ND
KP11	>256	>256	*aac (6)’-Ib*	>256	>256	ND
KP26	>256	>256	*aac (6)’-Ib*	128	>256	ND
KP63	>256	>256	*aac (6)’-Ib*	128	>256	ND
KP51	>256	>256	*aac (6)’-Ib*	128	>256	ND
**NDM and OXA-48-coproducing strain**						
KP1	32	16	*aac (6)’-Ib*	16	4	0.75
KP2	32	16	*aac (6)’-Ib*	4	8	0.63
KP5	32	16	*aac (6)’-Ib*	8	4	0.5
KP8	32	16	*aac (6)’-Ib*	16	4	0.75
KP12	32	16	*aac (6)’-Ib*	4	8	0.63
KP21	32	16	*aac (6)’-Ib*	8	4	0.5
KP30	32	16	*aac (6)’-Ib*	8	4	0.5
KP39	32	16	*aac (6)’-Ib*	16	4	0.75
KP81	32	16	*aac (6)’-Ib*	4	16	0.75
KP90	32	1	NF	8	0.25	0.5
KP36	128	>256	*aac (6)’-Ib*, *aphA6*, *armA*	32	128	ND
KP4	>256	16	*aac (6)’-Ib*	>256	8	ND
KP6	>256	16	*aac (6)’-Ib*	128	8	ND
KP15	>256	16	*aac (6)’-Ib*	>256	8	ND
KP18	>256	16	*aac (6)’-Ib*	>256	8	ND
KP19	>256	32	*aac (6)’-Ib*	>256	32	ND
KP7	>256	16	*aac (6)’-Ib*	32	8	ND

FICI: Fraction Inhibitory Concentration Index; NF: Not Found; ND: Not Determined

## Discussion

The occurrence of carbapenemase-producing *K*. *pneumoniae* has been globally mediated by carbapenemase productions (including KPC, OXA-48, NDM, IMP, and VIM). NDM and OXA-48-co-producing strains have been endemic especially in Asia including Thailand [1, 13, 14]. The emergence of carbapenem-resistant *K*. *pneumoniae* leads to limit treatment options and requires novel active agents or combination therapy. Fosfomycin has been proposed as an effective agent against multidrug-resistant *K*. *pneumoniae* as well as carbapenem-resistant *K*. *pneumoniae* [4, 15]. Our results demonstrated that 68.2% of carbapenemase-producing *K*. *pneumoniae* isolates were resistant to fosfomycin, while the resistance rate from worldwide is approximately 39.2%-66.2% [1]. These data suggest that fosfomycin monotherapy is unsuitable for being empirical therapy and requires susceptibility testing.

The major mechanism of fosfomycin resistance in Enterobacteriaceae is the production of fosfomycin modifying enzymes, particularly FosA, which catalyze fosfomycin [4]. The gene encoding FosA, called *fosA*^KP^ or *fosA5*, is present in 99.7% of *K*. *pneumoniae* genomes [16]. However, the intrinsic *fosA5* gene was found in both susceptible and resistant isolates suggesting that other mechanisms involve in fosfomycin resistance in *K*. *pneumoniae*. Plasmid-mediated *fosA3* which originates from the chromosome of *Kluyvera georgiana* mobilized to plasmid and spread in Enterobacteriaceae including *K*. *pneumoniae* resulting in fosfomycin resistance [17, 18]. Notably, 16 fosfomycin-susceptible isolates in our study exhibited silence of *fosA3*. Plasmid-mediated *fosA3* of these isolates elevated fosfomycin MICs of *E*. *coli* DH5α transformants supporting that fosfomycin monotherapy might be inappropriate for treatment of carbapenemase-producing *K*. *pneumoniae* infection. High prevalence of fosfomycin resistance in KPC-producing isolates from China is associated with plasmid co-harboring *fosA3* with *bla*_KPC_ [19]. Moreover, co-carriage of *fosA3* and *bla*_NDM_ has been found in *Salmonella enterica* serovar Corvallis in Germany [20] and plasmid co-carrying *fosA3* and an ESBL gene, *bla*_CTX-M_ has been reported in *E*. *coli* [3] indicating co-transfer of multiple resistance genes. Although *E*. *coli* DH5α transformants were selected on MHA supplemented with only fosfomycin, we also found *bla*_OXA-48_ in the transformants carrying *fosA3* indicating co-transfer of carbapenemase gene and *fosA3* (S2 Table). However, co-carriage of these resistance genes was not determined.

Apart from FosA production, fosfomycin resistance is also mediated by amino acid substitutions in fosfomycin target (MurA) and transporter proteins (GlpT and UhpT). The mutation at the active site (Cys115) of MurA has never been reported in *K*. *pneumoniae* including in our isolates indicating high fitness cost of the mutation [12, 21]. Thr287Asn substitution was found in both fosfomycin susceptible and resistant isolates suggesting that this substitution is unable to confer fosfomycin resistance. However, Thr287Asn in MurA was able to elevate fosfomycin MIC of *E*. *coli* DH5α transformant [12]. GlpT and UhpT, the transporter of G3P, and G6P, respectively in *K*. *pneumoniae* are also mediated fosfomycin influx and defective in these transporters involves in fosfomycin resistance [4, 7]. Therefore, impair growth of *K*. *pneumoniae* in M9 minimal medium supplemented with G3P or G6P indicates the defective transporter which related to fosfomycin resistance. In our study, many amino acid substitutions (Gly386Ser, Gly386Ile, Phe112Ser, and Pro97Arg) or loss of GlpT reduced growth of high-level fosfomycin-resistant isolates in the presence of G3P as a carbon source. The Arg171Vla substitution in UhpT was also detected in both fosfomycin-susceptible and fosfomycin-resistant isolates. The addition of Ala176Pro and Leu132Val substitutions were observed in high-level resistant isolates. Therefore, our results strongly suggest that FosA3 overexpression together with mutations or loss of GlpT or/and UhpT transporters are associated with high-level fosfomycin resistance in carbapenemase-producing *K*. *pneumoniae*.

Due to inadequate monotherapy, fosfomycin has been frequently combined with other active agents including carbapenems, colistin, and amikacin which exhibit synergism against carbapenem-resistant *K*. *pneumoniae* [8, 9, 22]. In our study, we focused on non-carbapenem and non-colistin, amikacin which has been reported to be an effective antibiotic against carbapenem-resistant *K*. *pneumoniae* [4]. Unfortunately, both amikacin-susceptible and amikacin-resistant isolates carried *aac (6)’-Ib* and *aphA6* which are common AME genes conferring amikacin resistance in *K*. *pneumoniae* [23, 24]. High-level amikacin-resistant carrying *armA* isolates have been co-carried with *bla*_KPC_, [25] and *bla*_CTX-M_ [26]. Although fosfomycin and amikacin combination has been demonstrated to be a potential combination against KPC-producing *K*. *pneumoniae* [22]. Unfortunately, synergism was rarely found in our study especially against isolates with high resistance to fosfomycin or/and amikacin. This discrepancy might be due to 1) different carbapenemases and *K*. *pneumoniae* strains, 2) co-carriage of multiple resistance genes and 3.) silence of resistance genes. However, these presumptions require further investigation.

In summary, the majority of carbapenem-resistant *K*. *pneumoniae* isolates are co-producing NDM and OXA-48 isolates. Overexpression of *fosA3* with the mutations of *glpT* (even loss of *glpT*) and/or *uhpT* are associated with high-level resistance to fosfomycin. The silence of *fosA3* frequently occurs in fosfomycin-susceptible *K*. *pneumoniae*. Amikacin cannot restore the activity of fosfomycin to achieve synergism against carbapenemase-producing *K*. *pneumoniae*, particularly isolates which are highly resistant to either fosfomycin or amikacin. Our results suggest that fosfomycin monotherapy may be inadequate due to the silence of *fosA3* and high resistance rate. Moreover, fosfomycin with amikacin may also be insufficient combination therapy against carbapenemase-producing *K*. *pneumoniae* isolates.

## Supporting information

S1 Table Oligonucleotide sequences of primers used in this study. (PDF)

S2 Table Characteristics of *K*. *pneumoniae* and *E*. *coli* DH5α transformants. (PDF)
