# Supplementary material for: fosA3 overexpression with transporter mutations mediates high-level of fosfomycin resistance and silence of fosA3 in fosfomycin-susceptible Klebsiella pneumoniae producing carbapenemase clinical isolates
Source: PLoS One. 2020 Aug 28;15(8):e0237474. doi: 10.1371/journal.pone.0237474 (PMC7454978; doi:10.1371/journal.pone.0237474)
Supplement: S1 Table — (PDF) [file pone.0237474.s001.pdf]

S1 Table. Oligonucleotide sequences of primers used in this study.

Gene	Primer sequences	PCR product size (bp)	Reference
fosA	F- ATCTGTGGGTCTGCCTGTCGT	271	Lu et al. [1]
	R- ATGCCCGCATAGGGCTTCT		
fosA3	F- CGGAGCCTATCTCTCCTGTG	219	this study
	R- CCGTCAGGGTCGAGAAAATA		
fosA5	F- GCGCCGAGCGTGGCGTTT	283	this study
	R- GCCATCGGGATCGAGGAA		
fosB	F- AGGTGAGACCTCGGCCTATT	302	this study
	R- GAGGTTTAGCCTCTTTATAATAACTCA		
fosC2	F- GGGCATATCTGAGCTTGGAG	212	this study
	R- CAATTTATGGCCGTCAGGAT		
fosX	F- GTTGCGTTTAAGGCAGGAAG	425	this study
	R- GGCTCCATTTGTTGGACAGT		

Reference

1. Lu PL, Hsieh YJ, Lin JE, Huang JW, Yang TY, Lin L, Tseng SP (2016) Characterisation of fosfomycin resistance mechanisms and molecular epidemiology in extended-spectrum beta-lactamase-producing *Klebsiella pneumoniae* isolates. Int J Antimicrob Agents 48 (5):564-568. doi:10.1016/j.ijantimicag.2016.08.013 PMID: 27765412
