# Supplementary material for: fosA3 overexpression with transporter mutations mediates high-level of fosfomycin resistance and silence of fosA3 in fosfomycin-susceptible Klebsiella pneumoniae producing carbapenemase clinical isolates
Source: PLoS One. 2020 Aug 28;15(8):e0237474. doi: 10.1371/journal.pone.0237474 (PMC7454978; doi:10.1371/journal.pone.0237474)
Supplement: S2 Table — (PDF) [file pone.0237474.s002.pdf]

S2 Table. Characteristics of *K. pneumoniae* and *E. coli* DH5α transformants.

Strains	Fos gene	Carbapenemase gene	MIC (mg/L)		
			Fosfomycin	Imipenem	Meropenem
KP23	*fosA3*, *fosA5*	*bla*$_{NDM}$	16	16	128
KP58	*fosA3*, *fosA5*	*bla*$_{NDM}$	>256	256	256
KP44	*fosA3*, *fosA5*	*bla*$_{OXA-48}$	32	4	16
KP51	*fosA3*, *fosA5*	*bla*$_{OXA-48}$	>256	8	32
KP5	*fosA3*, *fosA5*	*bla*$_{NDM}$, *bla*$_{OXA-48}$	32	32	64
KP4	*fosA3*, *fosA5*	*bla*$_{NDM}$, *bla*$_{OXA-48}$	>256	32	64
KP6	*fosA3*, *fosA5*	*bla*$_{NDM}$, *bla*$_{OXA-48}$	>256	32	64
KP15	*fosA3*, *fosA5*	*bla*$_{NDM}$, *bla*$_{OXA-48}$	>256	32	64
KP18	*fosA3*, *fosA5*	*bla*$_{NDM}$, *bla*$_{OXA-48}$	>256	256	256
KP19	*fosA3*, *fosA5*	*bla*$_{NDM}$, *bla*$_{OXA-48}$	>256	>256	256
KP7	*fosA3*, *fosA5*	*bla*$_{NDM}$, *bla*$_{OXA-48}$	>256	64	128
DH5α	No	No	0.5	0.25	0.015
DH5α/*fosA3*_KP23	*fosA3*	No	32	0.25	0.015
DH5α/*fosA3*_KP58	*fosA3*	No	128	0.25	0.015
DH5α/*fosA3*_KP44	*fosA3*	*bla*$_{OXA-48}$	16	0.25	0.015
DH5α/*fosA3*_KP51	*fosA3*	*bla*$_{OXA-48}$	>256	0.5	0.03
DH5α/*fosA3*_KP5	*fosA3*	*bla*$_{OXA-48}$	32	0.25	0.015
DH5α/*fosA3*_KP4	*fosA3*	*bla*$_{OXA-48}$	32	0.25	0.015
DH5α/*fosA3*_KP6	*fosA3*	*bla*$_{OXA-48}$	128	0.5	0.015
DH5α/*fosA3*_KP15	*fosA3*	*bla*$_{OXA-48}$	128	0.25	0.03
DH5α/*fosA3*_KP18	*fosA3*	*bla*$_{OXA-48}$	64	0.25	0.03
DH5α/*fosA3*_KP19	*fosA3*	*bla*$_{OXA-48}$	128	0.25	0.015
DH5α/*fosA3*_KP7	*fosA3*	*bla*$_{OXA-48}$	256	0.5	0.03
